# Influence of inter-proximal dimensions on inter-dental papilla presence

**DOI:** 10.6026/973206300210091

**Published:** 2025-01-31

**Authors:** Jinal Desai, Shivlal Vishnoi, Priyadarshini P Nadig, Kandarp Raj, Darshna Kasundra, Dhwani Bharucha

**Affiliations:** 1 Department of Periodontology, Manubhai Patel Dental College and Oral research Institute, Vadodadara, Gujarat, India

**Keywords:** Interdental papilla, aesthetics, embrasure

## Abstract

The presence of interdental papilla in relation to vertical distance between alveolar crest and the contact point, as well as horizontal distance between adjacent roots is of interest to dentists. Hence, 50 patients with a total of 501-papilla were examined. Following extensive debridement, mucoperiosteal flap was reflected and measurements of horizontal and vertical distances between adjacent roots were taken. Prior to reflection, each papilla was also given a JEMT index score. Linear trend analysis was done using χ^2^ test to examine the incidence of papilla and in 88.9% of cases, inter-dental papilla were found when vertical distances was 4 mm (P<0.01). Additionally, when horizontal distances were between 0.5 and 1 mm, papilla were present in 95.7% to 100% of cases (P<0.01). Thus, the presence of inter-proximal papilla was significantly influenced by vertical and horizontal distances of inter-proximal areas, both separately and in combination.

## Background:

The existence or absence interproximal papilla is a major concern for patients, periodontists and restorative dentists. Phonetic issues, lateral food impaction and cosmetic deformities can result from papilla loss [1]. In 1959, Cohen described the morphology of interdental papilla as gingival portion that occupies the space between adjacent teeth [2]. A space cervical to the interproximal contact is called a gingival embrasure [3] and it is open if the gingival papilla does not fill the embrasure space entirely. Open gingival embrasures also known as "black triangle," are a common problem in aging adult population, which is afflicted with periodontal disease. Alterations in the dimensions of papilla during orthodontic alignment, recession brought on by a loss of clinical attachment, a decrease in height of alveolar bone in relation to interproximal contact, size of embrasure area, angulations of root, location of interproximal contact and triangular crowns are the most likely causes [4]. The vertical and horizontal dimensions of interproximal areas significantly influenced the presence of interproximal papillae, both independently and in combination [5]. Before beginning dental treatment, patient should be informed about black triangles, which affect over one-third of adults [3].The interdental space is made up of horizontal measurements between mesial and distal surfaces of adjacent roots and vertical measurements between contact point and alveolar crest. Tarnow *et al.* [1] found that vertical dimension of interdental tissue between alveolar crest and contact point is less than 5 mm, interdental papilla is nearly always present. Tal [6] found that as the interproximal distance increases, the number of infra-bony pockets rises proportionately to the horizontal distance of interdental tissue. Many studies have looked at the presence of interdental papilla in relation to the distance between alveolar crest and contact point, but fewer studies have looked at the relationship between interproximal root distance and presence of interdental papilla [1, 7 and 8]. Therefore, it is of interest to assess the presence of interdental papilla in relation to horizontal distance between adjacent roots and also vertical distance between the contact point and alveolar crest.

## Methodology:

This single-center clinical observational study was planned and performed in Department of periodontics at Manubhai Patel Dental College and Hospital. It complies with the Helsinki Declaration and was accepted by the Institutional Ethical Committee. To ascertain minimum number of papillae required to optimize the outcomes, a power analysis was conducted. Approximately 250 papilla were required for the study, which included 501 interdental areas (184 anterior, 151 premolar and 166 molar areas) and this was necessary because the expected incidence of papilla was 50% with a power of 80% and a confidence level of 95%. The study included 50 patients in total with 24 men and 26 women. Every patient provided informed consent and all the patients underwent full thickness flap reflection. The sites included patients with periodontitis as defined by clinical attachment level > 4 mm and probing depth > 4 mm. In order to get the proper insights, nearby healthy areas had been opened and also releasing incisions were given. Scaling and root planing were performed two weeks prior to the surgery. The following conditions were completely ignored since they might have an impact on the presence of interdental papilla and health:

[1] Areas where periodontal surgery has previously been performed

[2] Regions with an exposed furcation (such as a rotated or interproximal concavities in a root)

[3] Regions without interdental contacts

[4] Regions that have received orthodontic treatment

[5] Teeth that are tilted or trans-located

[6] Any swollen or inflamed areas.

## Assessing the presence of interdental papilla:

In order to improve visibility, interdental papilla was dried and the affected area was isolated and each interdental area was closely examined in relation to interdental papilla for the identification of black triangle. It was then classified as: 1) papilla were deemed present when they completely fills the interdental space and 2) papilla were deemed absent when they do not cover inter dental space up to the contact point, resulting in the appearance of a black triangle beneath the contact point. Every interdental papilla also received a JEMT index score.

## Vertical distance between contact point and alveolar crest (VD):

Mucoperiosteal flap was raised after the administration of local anesthesia. After a full debridement process, vertical distances between the lowest point of contact surface of the teeth and alveolar crest closest to the occlusal side were measured. Any measurements that were less than 1 mm were rounded off using a graded Williams' periodontal probe. The most commonly measured and recorded numbers were used to generate final measurements with three measurements made at each site to reduce errors.

## Horizontal distance between adjacent roots (HD):

The interproximal distance between roots at the highest alveolar crest levels was calculated after vertical dimension was measured. A geometric divider was used to measure the separation between the mesial tooth's distal surface and the distal tooth's mesial surface. After that, the measurements had been transferred to a common measuring device and the outcome was recorded. Discrete ranges ranging from 0.5 to 4 mm were used to group measuring values, with each range being enlarged by 0.5 mm. One individual was permitted to take the measurements five times for each region in order to reduce measurement mistakes and the most commonly taken and recorded readings were chosen to calculate the final measurement.

## Statistical analysis:

Mean, standard deviation, frequencies and percentage distribution of the gathered data were ascertained by the examiner using descriptive statistics after entering the data into Microsoft Excel software. The association between papilla existence and vertical dimension and between papilla existence and horizontal dimension was evaluated using Pearson's Chi-Square. The association between the presence of papillae and the combined effect of vertical dimension and horizontal dimension was evaluated using the Pearson Correlation coefficient. Statistical analyses were performed using the Statistical Package for Social Sciences (SPSS) 16.0. A significance threshold of P < 0.01 was established.

## Results:

## Presence of interdental papilla in relation to vertical dimensions (VD):

The minimum number of sites showing vertical dimension was 10 mm, while maximum number of interproximal sites showing were 6 mm. If papilla are present, majority of them were concentrated between 4 and 6 mm vertical dimension. The findings showed that number of papilla present decreased steeply after 5 mm vertical dimension, indicating that 5 mm vertical dimension is essential for the presence of interdental papilla. Additionally, number of interdental papilla declined statistically significantly (P <0.01) as vertical dimensions increased after 5 mm vertical dimension (Table 1).

## Presence of interdental papilla in relation to horizontal dimensions (HD):

Minimum number of sites showing horizontal dimension was 4.5 mm, while maximum number of interproximal sites showing horizontal dimension was 2 mm. The majority of existing papilla was concentrated between 0.5 and 2 mm horizontal dimension, if we take their into account. The findings showed that number of papilla present decreased sharply after 1.5 mm horizontal dimension, indicating that 1.5 mm horizontal dimension is essential for the existence of interdental papilla. Additionally, number of interdental papilla that were already present decreased after 1.5 mm horizontal dimension and these results were statistically significant (P<0.01) (Table 2).

## Presence of interdental papilla in relation to horizontal distance and vertical distance:

**Figure 1:**
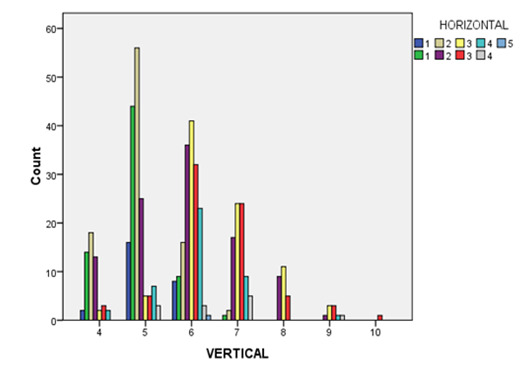
Distribution of papillae according to vertical and horizontal dimensions

The findings demonstrated that papilla were 100% present when horizontal distance was ≤ 1.5 mm and vertical distance was ≤ 5 mm. As the distance between alveolar crest and contact point increased, number of papilla that occupied interproximal space between adjacent roots decreased, adhering to the dimensions of 1.5* 5 mm. Additionally, if interproximal distance between roots are increased, the impact of a larger distance between alveolar crest and contact point become more apparent (Figure 1).

## Distribution of papilla in posterior and anterior segments:

On examining the incidence of papilla presence for posterior teeth independently, it was shown that an increase in vertical distance for horizontal dimensions of 5 mm considerably reduced the incidence of papilla presence (P <0.01). Similarly, when the incidence of papilla presence was considered independently for anterior teeth and incidence of papilla presence reduced dramatically for a horizontal distance as the vertical measurements are increased (P <0.01).

## Distribution of papillae according to JEMT's score for vertical dimensions:

**Figure 2:**
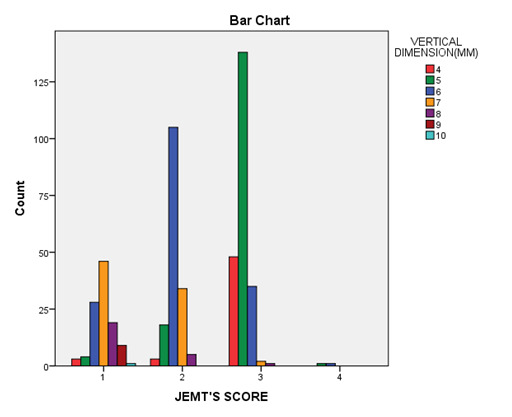
Distribution of papilla according to vertical distance and JEMT's score

The vertical dimension of 5 mm showed the highest number of papillae with score 3. The findings showed a negative correlation between the vertical dimension of papilla and JEMT's score assigned to each one after 5 mm. An increase in vertical distance between contact point and alveolar crest after 5 mm is associated with a lower score of JEMT's index with a less aesthetically pleasing appearance (Figure 2).

## Distribution of papilla according to JEMT's score for horizontal dimensions:

**Figure 3:**
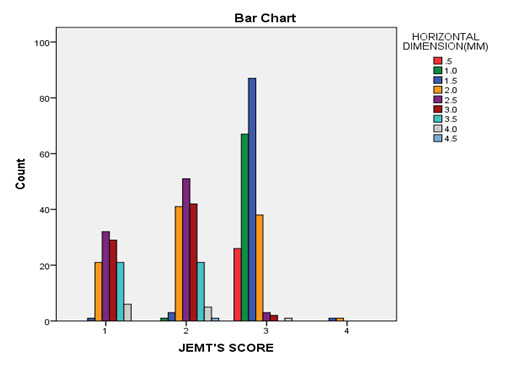
Distribution of papilla according to horizontal dimensions and JEMT's score

The horizontal dimension of 1.5 mm showed the highest number of papillae with score 3. The findings showed that, for each papilla after 1.5 mm, JEMT's score and the horizontal dimension of papilla had an inverse relationship. A lower-class number of JEMT's index and consequently, a less attractive appearance are associated with an increase in horizontal dimensions between adjacent roots and alveolar crest after 1.5 mm (Figure 3).

## Discussion:

The relationship between the hard and soft tissues surrounding the interdental papillae has been referred to as the "interdental papillae house." The interproximal space that contains interdental papilla is compared to a house and it comprises tooth surface, cement enamel junction (CEJ), contact area and the area where soft tissue and root meet above the alveolar bone crest [9]. Numerous factors including tooth alignment, structure of neighbouring tooth and inter-radicular bone influence the papillae, which are preserved in different forms. The fibers that pass through alveolar bone connecting teeth and gingiva are called interdental papilla and they support and shield the alveolar bone underneath and also keep the shape of dental arch against mechanical masticatory stress [3]. As demonstrated by Ko-Kimura *et al.* [3], 67% of people over 20 had open embrasures, compared to 18% of people under 20. The authors ascribed this discrepancy to aging-induced papilla height loss, keratinization decline and oral epithelium thinning. According to the literature, both vertical dimension of the interdental space and level of alveolar crests beneath the interdental space affect the presence of interdental papilla. Salama *et al.* [10] suggest that interdental distance may change the shape of interdental soft tissue and alveolar bone. Moscow *et al.* [11^11^] also observed that the quantity of papilla present decreases with increasing distance between alveolar crests and contact point.

The present study was carried out to investigate the presence of interdental papilla with respect to both horizontal distance between adjacent roots and vertical distance between the contact point and alveolar crest in order to gain a better understanding of their presence and embrasure space in general. Periodontal disease has been associated with the loss of interdental papilla due to the loss of bone. According to Tal [6], after periodontal surgery, fiber groups may rearrange or get severed, col may be removed beneath the interdental contacts and a scar may form in the interdental soft tissue. According to van der Velden [7], exposed furcation area may result in an abnormal shape of the interdental soft tissue, a propensity for inflammation and severe attachment loss. Takei [3] noted that it can be challenging to measure the contact points in regions where teeth are tilted or trans-positioned and that frequent inflammation from food impaction was expected to cause papilla denaturation. The exposed furcation (including a rotated root or one with interproximal concavities) and the area that had undergone periodontal surgery were excluded from this study due to the possibility of measurement errors. Areas that had received orthodontic treatment were also not included because it can reshape the interdental contact area, deform the interdental papilla and artificially suppress the interdental soft tissue. Every area that had any kind of inflammation was left out because the swelling could lead to errors of judgement. To identify the presence or absence of interdental papilla more accurately and thoroughly, the current study employed a classification system that considers the first-look aesthetics aspect of the entire smile unit. In 1997, Jemt [12] proposed this classification for the interdental soft tissue of adjacent implants. In the current study, this classification was related to natural teeth rather than implant sites, as it was intended. A full thickness flap was raised and alveolar crest was visualized to identify the actual distance between contact point and alveolar crest instead of using the blind technique. These techniques differ from those described in other studies that used various papilla selection processes and measuring techniques. We used a triangle scale to determine shortest interproximal distance between roots and it was useful because it could measure horizontal distance precisely by being positioned flat with occlusal side and inserted between mesial root surface of the distal tooth and the distal root surface of mesial tooth at alveolar crest level. The current study reveals a strong correlation between a decrease in the presence of interdental papilla with an increase in vertical distance between contact point and alveolar crest. Based on vertical distance between alveolar crest and contact point, presence of papillae was highest at 4 mm (88.9%), then it dropped to 36.0% at 6 mm and then it further declined as the vertical distance increased. These findings concur with those of other Ryser *et al.* studies [13].

## Conclusion:

The existence of inter-proximal papilla is therefore significantly influenced by vertical and horizontal dimensions of inter-proximal areas, both independently and in combination. Additionally, a JEMT's index can be a helpful tool for accurately determining whether the interdental papilla is present or absent and its scores closely matches the aesthetic value of inter-dental papilla among other parameters. Therefore, by preserving vertical and horizontal dimensions of interdental space, it is possible to achieve both predictability of the inter-papillary dimensions and an aesthetically pleasing result. This fact should be taken into account during orthodontic procedures as well as when restoring natural teeth or tooth-implant restorations.

## Figures and Tables

**Table 1:** Presence of interdental papilla in relation to vertical distance between contact point and alveolar crest

**Vertical Distance Between Contact Point and Alveolar Crest (mm)**								
	4	5	6	7	8	9	10	TOTAL
Total Number of Inter-proximal Sites	54	161	169	82	25	9	1	501
Papilla Present(n)	48	139	36	2	1	0	0	226
(%)	-88.90%	-86.30%	-21.30%	-2.40%	-4%	0%	0%	-45.10%
Papillae Absent(n)	6	22	133	80	24	9	1	275
(%)	-11.10%	-13.70%	-78.70%	-97.60%	-96%	-100%	-100%	-54.90%

**Table 2:** Presence of interdental papilla according to horizontal distance between adjacent roots

**Horizontal Distance Between Adjacent Roots (mm)**										
	0.5	1	1.5	2	2.5	3	3.5	4	4.5	TOTAL
Total Number of Inter-proximal Sites	26	68	92	101	86	73	42	12	1	501
Papilla Present	26	67	88	39	3	2	0	1	0	226
(%)	100%	98.50%	95.70%	38.60%	3.50%	2.70%	0%	8.30%	0%	-45.10%
Papilla Absent	0	1	4	62	83	71	42	11	1	275 (54.9%)
(%)	0%	1.50%	4.30%	61.40%	96.50%	97.30%	100%	91.70%	100%	
